# Localised Badger Culling Increases Risk of Herd Breakdown on Nearby, Not Focal, Land

**DOI:** 10.1371/journal.pone.0164618

**Published:** 2016-10-17

**Authors:** Jon Bielby, Flavie Vial, Rosie Woodroffe, Christl A. Donnelly

**Affiliations:** 1 Institute of Zoology, Regent’s Park, London, NW1 4RY, United Kingdom; 2 Epi-Connect, Djupdalsvägen 7, 14251, Skogås, Sweden; 3 MRC Centre for Outbreak Analysis and Modelling, Department of Infectious Disease Epidemiology, Faculty of Medicine, Imperial College, London, W2 1PG, United Kingdom; Universidade de Aveiro, PORTUGAL

## Abstract

Bovine tuberculosis is an important disease affecting the UK livestock industry. Controlling bovine tuberculosis (TB) is made more complex by the presence of a wildlife host, the Eurasian badger, *Meles meles*. Repeated large-scale badger culls implemented in the Randomised Badger Culling Trial (RBCT) were associated with decreased cattle risks inside the culling area, but also with increased cattle risks up to the 2km outside the culling area. Intermediate reductions in badger density, as achieved by localised reactive culling in the RBCT, significantly increased cattle TB. Using a matched-pairs case-control study design (n = 221 pairs of cattle herds), we investigated the spatial scale over which localised badger culling had its biggest impact. We found that reactive badger culling had a significant positive association with the risk of cattle TB at distances of 1-3km and 3-5km, and that no such association existed over shorter distances (<1km). These findings indicate that localised badger culls had significant negative effects, not on the land on which culling took place, but, perhaps more importantly, on adjoining lands and farms.

## Introduction

Bovine tuberculosis (TB), caused by *Mycobacterium bovis*, is an expensive problem for British cattle-farmers and tax-payers. Regular tuberculin testing of cattle herds is a key component of efforts to control cattle TB and limit its spread. Herds that are confirmed as harbouring *M*. *bovis* infection are placed under temporary movement restrictions and those cattle within the herd that test positive are slaughtered (an event now known as an Officially Tuberculosis Free status Withdrawn (OTF-W) herd breakdown). In 2014 more than 8 million cattle tests were conducted in Great Britain and, as a consequence, there were over 4000 new herd incidents of TB, almost 3000 of which resulted in OTF status being withdrawn [1]. The annual cost of managing TB in cattle is considerable, approaching £100 million per year [2]. Control efforts are complicated by the presence of a wildlife host, the Eurasian badger (*Meles meles*), which has been implicated in the transmission of TB to cattle [3–5].

The role of badgers as a source of *M*. *bovis* infection in cattle resulted in badger culling having been a component of the varied policies aimed at controlling cattle TB since the early 1970s [3,5]. However, because these approaches failed to prevent the spread of cattle TB, a large-scale field study, the Randomised Badger Culling Trial (RBCT), was initiated in 1998 to measure the effectiveness of badger culling as a means of reducing cattle TB.

Within the RBCT, localised (“reactive”) culling was shown to increase the incidence of confirmed (now termed OTF-W) TB herd breakdowns relative to herds on comparable unculled survey-only lands [6]. Localised culling, undertaken in response to specific confirmed herd breakdowns and conducted across badger territories which overlapped each breakdown farm (including adjoining land), reduced badger density in reactive trial areas by approximately 30% relative to unculled survey-only trial areas (as measured by the density of badger field signs estimated through field surveys of at least 20km^2^ of accessible land per RBCT area [7]). This localised culling led to an increased confirmed herd breakdown incidence of 27% relative to cattle herds in unculled survey-only RBCT areas (95% confidence interval: 4.8–53% increase, whereas when the confidence interval was corrected using an inflation factor to account for overdispersion it broadened from a 2.4% decrease to a 65% increase in herd breakdown incidence; [6]). This increase in confirmed herd breakdown incidence appeared to occur because reductions in badger density led to changes in badger spatial organisation. These changes resulted in increased ranging and dispersal [8,9], elevated the prevalence of TB infection among badgers [10], and potentially caused an increase in the number of infectious contacts between badgers and nearby cattle herds.

In addition to localised RBCT culling being associated with increased TB risks for cattle on land randomised to receive reactive culling [6,11], a similar increase was observed among cattle herds on unculled land adjoining repeated wide-scale (“proactive”) RBCT culling areas [4]. The scale at which such epidemiological effects occur is likely to be context-dependent, but detailed study of pre-RBCT localised culls suggested that social perturbation may occur one or more social group territories distance from culled groups [12]. Associations between localised RBCT badger culling and *M*. *bovis* infection/confirmed herd breakdowns have been previously been shown to exist up to a distance of 5km from culled land [11] and changes to badger dispersal in response to culling may reach and even exceed this distance on occasion [8].

Given that cattle herds occupy land within a network of largely contiguous, adjoining farms, it is important to understand the spatial scale over which localised culling may affect risks to nearby cattle, that is, how risk of breakdown varies as a function of the distance from the culled land. Thus, studying the spatial epidemiological patterns can provide an improved understanding of infection spread and inform efforts to limit the transmission of *M*. *bovis* from badgers to cattle.

To investigate the importance of the distance between badger culls and the risk of breakdown in surrounding herds, we undertook a matched-pair case-control study, similar to that described in Vial and Donnelly 2012 [11]. The earlier analyses focussed on addressing the question: Over what cumulative spatial-scale does localised culling impact on risk of herd breakdown (i.e. <1km; <3km; <5km)? Here, our aim is to identify, within the 5km range (the largest spatial scale included in Vial and Donnelly 2012 [11]), the distance category(ies) over which localised culling has its largest effect on risk of herd-breakdown (i.e. <1km, 1-3km, 3-5km from the culled land). Thus, we analysed the same data as Vial and Donnelly, but partitioned the data into a circle and two non-overlapping annuli (<1km, 1-3km, 3-5km from the culled land) to gain further insight into the small-scale spatial dynamics of culling-associated cattle TB risks.

## Methods

Herd breakdowns were defined as confirmed (OTF-W) if *M*. *bovis* was cultured from an individual within the herd, if *post-mortem* examination led to the discovery of visible lesions, or if routine slaughterhouse inspections of meat from a herd led to the discovery of TB lesions and subsequent culture of *M*. *bovis*. Each case (a confirmed herd breakdown) within a reactively culled RBCT area was paired with a control herd selected randomly from herds within the same RBCT area. To be considered a ‘control herd’, a herd must have experienced a clear herd test within a year of the herd breakdown in its pair-matched case herd and have no shared lands within 5km of the reactor land (‘reactor land’ being the land used by the breakdown herd). We focussed on confirmed herd breakdowns within the period of time from the first reactive cull until the suspension of reactive culling in November 2003. This data treatment led to the inclusion of 221 cases and their matched controls, of which 192 occurred in 2002 or later (for analysed dataset and data descriptors please see S1 and S2 Files). Note that the earlier Vial and Donnelly (2012) paper [11] included some additional breakdowns taking place before the first reactive cull and after its suspension, thus their sample size was larger.

As in Vial and Donnelly 2012 we set 5km as the maximum distance to investigate associations between localised badger culling and the risk of subsequent confirmed herd breakdowns because the majority of increased badger movements in response to RBCT culling were shorter than this distance [8]. For each matched case-control pair we calculated the number of badgers reactively culled within three non-overlapping zones: within a circle of radius 1km; within an annulus of radius 1 to 3km; and within an annulus of radius 3 to 5km. We compared the odds ratios (ORs) between these zones to quantify how risk of confirmed herd breakdowns varied as a function of distance from localised badger culling.

In addition to the number of badgers culled, for each matched case-control pair we also quantified, within each circle or annulus, a number of other herd risk factors, including local risk factors that have previously been identified as being important [13]. The variables analysed were: the number of confirmed herd breakdowns in the previous year; whether the case/control herd was a dairy herd; the size of the herd; the area of the farm; the historic incidence of confirmed herd breakdowns; and the number of tested unrestricted herds. A description of each follows.

To account for geographic variation in risk of confirmed herd breakdowns, we included variables quantifying, for each circle or annulus: the number of confirmed herd breakdowns in the past year or two years and the number of tested herds not under TB-related movement restrictions. In addition, the historic incidence of confirmed herd breakdowns for each case and each control herd was calculated for the three years prior to the initial proactive cull within the matched proactively culled area (except in triplets* D, I and J where it was calculated for the three years prior to the start of the 2001 foot-and-mouth disease epidemic; *triplets were matched sets of three RBCT areas one randomised to proactive culling, one to reactive culling and one to no culling, referred to as “survey-only”).

Herd-level data (herd type (dairy/non-dairy), herd size, and farm area) were also obtained for each herd within a matched case-control pair and included as covariates. By way of a sensitivity analysis, all continuous, spatially-variable data (the number of badgers culled, the number of confirmed breakdowns, and the number of tested herds not under TB-related movement restrictions) were calculated for one- and two-year windows prior to the date of breakdown detection in the case and the herd test date of the control. Following Vial and Donnelly 2012, we focussed our presentation of results and subsequent interpretation upon the one-year time window and offer for comparison the results from analysis of the data from the two-year window. Because the area of each zone increased with the distance from the case/control, we also conducted parallel analyses using the number of badgers culled per km^2^ as our predictor variable and compared results with those obtained from the raw data.

The Defra VetNet system provided data on herd breakdown history and herd-level variables that were included as covariates in the analyses. Continuous variables were log transformed before analysis. We used conditional logistic regression models to ascertain how the relationship between the number of badgers culled and the risk of confirmed herd breakdowns varied as a function of the distance from localised badger culling after adjusting for covariates. We report here the results from the multivariable models including all covariates (i.e. we adjusted for variables identified previously as epidemiologically relevant and did not drop any to simplify the model). As in Vial and Donnelly 2012, the estimated ORs and the confidence intervals reported relate to the change in cattle herd TB risk that would be observed with a doubling of that covariate. To do so, we used the following formulae:

OR = exp(log odds ratio * ln(2))

and the confidence limits were calculated using:

exp(log odds ratio * ln(2) ± 1.96*SE*ln(2))

where SE is the standard error of the estimated log odds ratio.

The exception was the categorical variable coding for the herd type (dairy/non-dairy), for which the OR and confidence intervals are presented showing the change in risk if a herd is dairy compared to if it were non-dairy.

## Results

Each of the three models incorporating data from only a single circle or annulus (i.e., <1km, 1-3km, or 3-5km) demonstrated a strong association between the number of badgers culled in the previous year and the risk of confirmed herd breakdown, even after other covariates had been accounted for (Summary of data values in Table 1; Results of analyses in Table 2). A doubling in the number of badgers culled within a 1km radius of the focal herd was associated with an increase in odds of breakdown of 28% (OR: 1.28, 95% CI: 1.14–1.44, p<0.001), as previously reported by Vial and Donnelly [11]. Going beyond these earlier analyses, a doubling of the number of badgers culled within a 1-3km annulus and within a 3-5km annulus of the herd was associated with increases of 45% (OR: 1.45, 95% CI: 1.27–1.66, p<0.001) and 46% (OR: 1.46, 95% CI: 1.27–1.69, p<0.001), respectively.

**Table 1:** Mean number of badger culled in the previous year, number of confirmed herd breakdowns in the previous year, and number of tested unrestricted herds in each circle or annulus for case and control herds.

	Distance (km)	Case herds	Control herds
Mean number of badgers culled in the previous year	<1	9.19	3.49
	1–3	22.82	8.01
	3–5	22.43	12.34
Mean number of confirmed herd breakdowns in the previous year	<1	4.38	3.19
	1–3	7.95	7.34
	3–5	10.84	10.49
Mean number of tested unrestricted herds	<1	7.82	6.31
	1–3	19.85	17.19
	3–5	28.80	26.10

**Table 2:** Results of models including only a single circle or annulus with the aim of investigating associations between herd-breakdowns and the number of badgers culled within the RBCT within the previous year, while adjusting for confirmed herd breakdowns and other herd-levels covariates. Results are presented of analyses based on data quantified, where appropriate, within a 1km radius, a 1-3km annulus, and a 3-5km annulus of the case-control. Estimated odds ratios and their confidence intervals correspond to the change in risk of herd breakdown associated with a doubling of that variable.

	Odds ratio (95% confidence interval); p-value		
Variable	<1km*	1-3km	3-5km
**Number of badgers culled in the previous year**	**1.28 (1.14–1.44); <0.001**	**1.45 (1.27–1.66); <0.001**	**1.46 (1.27–1.69); <0.001**
Number of confirmed herd breakdowns in the previous year.	1.24 (0.96–1.60); 0.097	0.84 (0.57–1.25); 0.402	0.71 (0.46–1.10); 0.131
Dairy herd	1.98 (1.08–3.72); 0.030	2.31 (1.23–4.35); 0.009	2.46 (1.29–4.72); 0.007
Herd size	0.97 (0.86–1.10); 0.642	0.95 (0.85–1.09); 0.538	0.96 (0.85–1.08); 0.509
Farm area	23.57 (7.66–68.07); <0.001	21.14 (6.88–64.99); <0.001	31.03 (9.51–101.23); <0.001
Confirmed historic incidence	0.94 (0.51–1.74); 0.844	0.87 (0.44–1.71); 0.681	0.79 (0.40–1.54); 0.477
Number of tested, unrestricted herds in the previous year	0.83 (0.65–1.06); 0.131	0.80 (0.58–1.11); 0.187	0.87 (0.64–1.20); 0.403
Negative log likelihood	103.63	97.75	100.54
Degrees of freedom	213	213	213

*These results were previously published by Vial and Donnelly (2012) in Table 2.

When models were constructed including data at all three distances simultaneously (thus not allowing the effects of other covariates to vary between models), the significant association between the number of badgers culled within 1km of the herd and risk of confirmed herd breakdowns disappeared (OR for a doubling of the number of badgers culled: 1.03, 95% CI: 0.87–1.22, p = 0.75, Table 3). However, the significant associations remained between badgers culled within the 1-3km and 3-5km annuli of the herd and the risk of confirmed herd breakdown. A doubling of the number of badgers culled within 1-3km increased the odds of herd breakdown by 24% (OR: 1.24, 95% CI: 1.01–1.50, p-value: 0.035), and a doubling between 3-5km similarly increased the odds by 27% (OR: 1.27, 95% CI: 1.07–1.50, p-value: 0.006).

**Table 3:** Results of models investigating associations between herd-breakdowns and the number of RBCT culled badgers within the previous year, including data at all three distances simultaneously, while adjusting for confirmed herd breakdowns and other herd-levels covariates. Estimated odds ratios and their confidence intervals correspond to the change in risk of herd breakdown associated with a doubling of that variable. Negative log likelihood of the model = 91.22, d.f. = 207.

Variable	Odds ratio (95% confidence interval); p-value
Number of badgers culled <1km in the previous year	1.03 (0.87–1.22); 0.750
**Number of badgers culled 1-3km in the previous year**	**1.24 (1.01–1.50); 0.035**
**Number of badgers culled 3-5km in the previous year**	**1.27 (1.07–1.50); 0.006**
Number of confirmed herd breakdowns <1km in the previous year	1.29 (0.97–1.72); 0.085
Number of confirmed herd breakdowns 1-3km in the previous year	0.84 (0.54–1.31); 0.446
Number of confirmed herd breakdowns 3-5km in the previous year	0.76 (0.45–1.26); 0.283
Dairy herd	2.48 (0.42–14.79); 0.009
Herd size	0.95 (0.84–1.09); 0.489
Farm area	29.22 (8.49–100.56); <0.001
Confirmed historic incidence	0.80 (0.38–1.68); 0.563
Number of tested, unrestricted herds <1km in the previous year	0.85 (0.61–1.18); 0.339
Number of tested, unrestricted herds 1-3km in the previous year	0.85 (0.50–1.45); 0.557
Number of tested, unrestricted herds 3-5km in the previous year	1.04 (0.63–1.70); 0.891

Quantifying variables over a time window of two years prior to the breakdown/date of clear test yielded similar results (S1 and S2 Tables), as did the results of the analyses taking into account the geographic area (i.e. the size in km^2^) of each circle or annulus over which badger culling was calculated (S3 and S4 Tables).

## Discussion

Our results provide evidence that in response to localised badger culls the risk of confirmed herd breakdown was significantly increased on farms between 1 and 5km from reactively culled lands. The estimated ORs corresponding to a doubling in the total number of RBCT badgers culled within 1-3km and 3-5km were nearly identical (1.24 and 1.27, respectively when all data were analysed simultaneously) suggesting that the number of badgers culled within these distances similarly affected the risk of confirmed herd breakdowns. Thus, the detrimental effects of localised culling upon cattle TB risk appeared to increase herd breakdown incidence on nearby, not focal, land. Our finding that risk of confirmed breakdowns is increased in neighbouring rather than culled land gives more precision than earlier research identifying that the negative consequences of localised culling could extend as far as 5km, without identifying within that range the distances over which culling most increased the risk of herd breakdown [11].

The distance over which herds experienced the greatest increased risk of confirmed herd breakdown risk was consistent with the spatial scale of changes to badger dispersal in response to culling. RBCT data suggest that, on average, dispersal events alter by a relatively short distance with the first one or two proactive culls (mean increase in dispersal at second cull = 640m [8]). However, with repeated culls the dispersal distances increase, with larger-scale events of up to and over 5km becoming much more common ([8, 14]. These data on the magnitude of changes to badger movement, combined with the increase in badger infection prevalence observed in response to localised reactive culling [10], suggest that post-culling increases in badger movement could play a role in transmission of TB at distances of up to 5km.

Consistent with our findings based on localised reactive cull areas, data from the RBCT collected on land subjected to repeated large-scale (proactive) culls suggested that increases in herd incidence were observed on adjoining land, rather than on focal land [4]. While a significant increase in incidence rate occurred among herds within 2km the culling treatment boundary, the estimated increase was reduced when herds up to 3km from the treatment boundary were included in the analyses. Moreover, while we found no evidence that small-scale culling within 1km of a farm location influenced subsequent breakdown risk (either positively or negatively), large-scale culling reduced breakdown risk in the culled areas [4]. Taken together these results highlight how different culling methodologies (e.g. proactive and reactive culling), and the associated reductions in host density that they deliver [7], may have different effects on badger social organisation, movement, risk of *M*. *bovis* transmission, and herd breakdown. It is therefore important to consider how the efficacy of a management tool such as culling may be affected by key differences in methodology.

The scale of increase in confirmed herd breakdowns prompted by reactive culling (27% [6]), and the distance over which this increased risk occurred (between 1 and 5km), highlight how relatively localised, moderate reductions to badger density can lead to landscape-level effects of considerable size. Regardless of the precise cattle- and badger-related mechanisms underpinning these increases, our results suggest that the detrimental impacts of localised reactive culling may be felt over a considerable spatial scale. This finding highlights the importance of considering the broader impacts within and upon the farmed landscape when considering implementing culling as a method of controlling the spread of bovine TB.

## Supporting Information

S1 File Dataset used for the analyses within this paper. (CSV)

S2 File Data descriptor of the dataset used for the analyses within this paper. (DOCX)

S1 Table Results of models including only a single circle or annulus with the aim of investigating associations between herd-breakdowns and the number of badgers culled within the RBCT within the previous two years, while adjusting for confirmed herd breakdowns and other herd-level covariates. Results are presented of analyses based on data quantified, where appropriate, within a 1km radius, a 1-3km annulus, and a 3-5km annulus of the case-control. Estimated odds ratios and their confidence intervals correspond to the change in risk of herd breakdown associated with a doubling of that variable.
(DOCX)

S2 Table Results of models investigating associations between herd-breakdowns and the number of RBCT culled badgers within the previous two years, including data at all three distances simultaneously, while adjusting for confirmed herd breakdowns and other herd-levels covariates. Estimated odds ratios and their confidence intervals correspond to the change in risk of herd breakdown associated with a doubling of that variable. Negative log likelihood of the model = 91.11, d.f. = 207.
(DOCX)

S3 Table Results of models including only a single circle or annulus with the aim of investigating associations between herd-breakdowns and the number of badgers culled per km^2^ within the RBCT within the previous year, while adjusting for confirmed herd breakdowns and other herd-level covariates. Results are presented of analyses based on data quantified, where appropriate, within a 1km radius, a 1-3km annulus, and a 3-5km annulus of the case-control. Estimated odds ratios and their confidence intervals correspond to the change in risk of herd breakdown associated with a doubling of that variable.
(DOCX)

S4 Table Results of models investigating associations between herd-breakdowns and the number of RBCT culled badgers per km^2^ within the previous year, including data at all three distances simultaneously, while adjusting for confirmed herd breakdowns and other herd-levels covariates. Estimated odds ratios and their confidence intervals correspond to the change in risk of herd breakdown associated with a doubling of that variable. Negative log likelihood of the model = 90.68, d.f. = 207.
(DOCX)

## References

[1] Department for Environment Food and Rural Affairs (2015) Bovine TB statistics https://www.gov.uk/government/policies/bovine-tuberculosis-bovine-tb.

[2] Department for Environment Food and Rural Affairs (2014) The Strategy for achieving Officially Bovine Tuberculosis Free status for England. https://www.gov.uk/government/uploads/system/uploads/attachment_data/file/300447/pb14088-bovine-tb-strategy-140328.pdf

[3] Krebs JR, Anderson R, Clutton-Brock T, Morrison I, Young D, Donnelly C, et al (1997) Bovine tuberculosis in Cattle and Badgers. H.M.S.O., London.

[4] Donnelly CA, Woodroffe R, Cox D, Bourne FJ, Cheeseman C, Clifton-Hadley RS, et al (2006) Positive and negative effects of widespread badger culling on tuberculosis in cattle. Nature 439: 843–846. 10.1038/nature04454 16357869

[5] Bourne J, Donnelly CA, Cox DR, Gettinby G, McInerney JP, Morrison WI, et al (2007) Bovine TB: The Scientific Evidence, Defra, London.

[6] Donnelly CA, Woodroffe R, Cox D, Bourne J, Gettinby G, Le Fevre AM, et al (2003) Impact of localized badger culling on tuberculosis incidence in British cattle. Nature 426: 834–837. 10.1038/nature02192 14634671

[7] Woodroffe R, Gilks P, Johnston W, Le Fevre A, Cox D, Donnelly C, et al (2008) Effects of culling on badger abundance: implications for tuberculosis control. Journal of Zoology 274: 28–37.

[8] Pope LC, Butlin RK, Wilson GJ, Woodroffe R, Erven K, Conyers CM, et al (2007) Genetic evidence that culling increases badger movement: implications for the spread of bovine tuberculosis. Molecular Ecology 16. 10.1111/j.1365-294X.2007.03553.x 17944854

[9] Woodroffe R, Donnelly CA, Cox D, Bourne F, Cheeseman C, Delahay R, et al (2006) Effects of culling on badger Meles meles spatial organization: implications for the control of bovine tuberculosis. Journal of Applied Ecology 43: 1–10.

[10] Woodroffe R, Donnelly CA, Cox D, Gilks P, Jenkins HE, Johnston WT, et al (2009) Bovine tuberculosis in cattle and badgers in localized culling areas. Journal of Wildlife Diseases 45: 128–143. 10.7589/0090-3558-45.1.128 19204342

[11] Vial F, Donnelly CA (2012) Localized reactive badger culling increases risk of bovine tuberculosis in nearby cattle herds. Biology Letters 8. 10.1098/rsbl.2011.0554 PMC3259956 21752812

[12] Tuyttens F, Delahay R, Macdonald D, Cheeseman C, Long B, Donnelly C (2000) Spatial perturbation caused by a badger (Meles meles) culling operation: implications for the function of territoriality and the control of bovine tuberculosis (Mycobacterium bovis). Journal of Animal Ecology 69: 815–828. 29313991 10.1046/j.1365-2656.2000.00437.x

[13] Vial F, Johnston WT, Donnelly CA (2011) Local cattle and badger populations affect the risk of confirmed tuberculosis in British cattle herds. PLoS ONE 6: e18058 10.1371/journal.pone.0018058 21464920 PMC3065452

[14] Department for Environment Food and Rural Affairs (2006) Research Project Final Report: Project Code SE3110, Molecular genetic analysis of badger social structure and bovine TB. http://randd.defra.gov.uk/Document.aspx?Document=SE3110_5295_FRP.doc.

