# Supplementary material for: Localised Badger Culling Increases Risk of Herd Breakdown on Nearby, Not Focal, Land
Source: PLoS One. 2016 Oct 17;11(10):e0164618. doi: 10.1371/journal.pone.0164618 (PMC5066978; doi:10.1371/journal.pone.0164618)
Supplement: S2 File — (DOCX) [file pone.0164618.s002.docx]

**Supplement**

**Localised Badger Culling Increases Risk of Herd Breakdown on Nearby, not Focal, Land.**

Jon Bielby^1^, Flavie Vial^2^, Rosie Woodroffe^1^, Christl A. Donnelly^3*^

^1^Institute of Zoology, Regent’s Park, London, NW1 4RY, UK

^2^Epi-Connect, Djupdalsvägen 7, 14251 Skogås, Sweden

^3^MRC Centre for Outbreak Analysis and Modelling, Department of Infectious Disease Epidemiology, Faculty of Medicine, Imperial College, London W2 1PG, UK

*Corresponding author

E-mail c.donnelly@imperial.ac.uk

**Dataset variable descriptors**

All variables below represent the data analysed after subtraction of the control data from the case data (i.e. case variable minus control variable) for each of the matched case-control pairs included in our analyses.

*The log transformed variables were of the form ln(x+0.5). The 0.5 allowed the transformation for all non-negative values of x.

Variable name Definition

ObjectID Unique ID of the case-control pair

Outcome Response variable (it equals 1 for each case-control pair)

Dairy Difference between the case and control herds in the dairy status of the herd (dairy = 1; non-dairy = 0)

Diffnumbadger1_1yr Difference between the case and control herds in the log transformed* number of badgers culled within a distance of 1km in the year prior to the case confirmed herd breakdown.

Diffnumbadger13_1yr Difference between the case and control herds in the log transformed* number of badgers culled at a distance of 1-3km in the year prior to the case confirmed herd breakdown.

Diffnumbadger35_1yr Difference between the case and control herds in the log transformed*number of badgers culled at a distance of 3-5km in the year prior to the case confirmed herd breakdown.

Diffnumbadger1_2yr Difference between the case and control herds in the log transformed*number of badgers culled within a distance of 1km in the 2 years prior to the case confirmed herd breakdown.

Diffnumbadger13_2yr Difference between the case and control herds in the log transformed* number of badgers culled at a distance of 1-3km in the 2 years prior to the case confirmed herd breakdown.

Diffnumbadger35_2yr Difference between the case and control herds in the log transformed* number of badgers culled at a distance of 3-5km in the 2 years prior to the case confirmed herd breakdown.

Diffclosenumbreaks1_1yr Difference between the case and control herds in the log transformed* number of confirmed herd breakdowns within a distance of 1km in the year prior to the case confirmed herd breakdown.

Diffclosenumbreaks13_1yr Difference between the case and control herds in the log transformed* number of confirmed herd breakdowns at a distance of 1-3km in the year prior to the case confirmed herd breakdown.

Diffclosenumbreaks35_1yr Difference between the case and control herds in the log transformed* number of confirmed herd breakdowns at a distance of 3-5km in the year prior to the case confirmed herd breakdown.

Diffclosenumbreaks1_2yr Difference between the case and control herds in the log transformed* number of confirmed herd-breakdowns within a distance of 1km in the 2 years prior to the case confirmed herd breakdown.

Diffclosenumbreaks13_2yr Difference between the case and control herds in the log transformed* number of confirmed herd-breakdowns at a distance of 1-3km in the 2 years prior to the case confirmed herd breakdown.

Diffclosenumbreaks35_2yr Difference between the case and control herds in the log transformed* number of confirmed herd-breakdowns at a distance of 3-5km in the 2 years prior to the case confirmed herd breakdown.

Diffherdsize Difference between the case and control herds in the log transformed* number of cattle in the herd (herd size).

Diffarea_km Difference between the case and control herds in the log transformed* area of the farm (in km^2^).

Diffprevbreak_1yr Difference between the case and control herds in the log transformed* number of confirmed herd breakdowns in the year prior to the case confirmed herd breakdown.

Diffprevbreak_2yr Difference between the case and control herds in the log transformed* number of confirmed herd breakdowns in the 2 years prior to the case confirmed herd breakdown.

DiffclosenumNR1_1yr Difference between the case and control herds in the log transformed* number of tested cattle herds not under TB-related movement restrictions within a distance of 1km in the year prior to the case confirmed herd breakdown.

DiffclosenumNR13_1yr Difference between the case and control herds in the log transformed* number of tested cattle herds not under TB-related movement restrictions at a distance of 1-3km in the year prior to the case confirmed herd breakdown.

DiffclosenumNR35_1yr Difference between the case and control herds in the log transformed* number of tested cattle herds not under TB-related movement restrictions at a distance of 3-5km in the year prior to the case confirmed herd breakdown.

DiffclosenumNR1_2yr Difference between the case and control herds in the log transformed* number of tested cattle herds not under TB-related movement restrictions within a distance of 1km in the 2 years prior to the case confirmed herd breakdown.

DiffclosenumNR13_2yr Difference between the case and control herds in the log transformed* number of tested cattle herds not under TB-related movement restrictions at a distance of 1-3km in the 2 years prior to the case confirmed herd breakdown.

DiffclosenumNR35_2yr Difference between the case and control herds in the log transformed* number of tested cattle herds not under TB-related movement restrictions at a distance of 3-5km in the 2 years prior to the case confirmed herd breakdown.
