# Supplementary material for: Localised Badger Culling Increases Risk of Herd Breakdown on Nearby, Not Focal, Land
Source: PLoS One. 2016 Oct 17;11(10):e0164618. doi: 10.1371/journal.pone.0164618 (PMC5066978; doi:10.1371/journal.pone.0164618)
Supplement: S1 Table — Results are presented of analyses based on data quantified, where appropriate, within a 1km radius, a 1-3km annulus, and a 3-5km annulus of the case-control. Estimated odds ratios and their confidence intervals correspond to the change in risk of herd breakdown associated with a doubling of that variable. (DOCX) [file pone.0164618.s003.docx]

|  | Odds ratio (95% confidence interval); p-value | | |
| --- | --- | --- | --- |
| Variable | <1km* | 1-3km | 3-5km |
| **Number of badgers culled in the previous two years** | **1.12 (1.00-1.25);**  **0.044** | **1.38 (1.22-1.57); <0.001** | **1.52 (1.30-1.790); <0.001** |
| Number of confirmed herd breakdowns in the previous two years | 1.53 (1.10-2.13); 0.012 | 0.91 (0.58-1.42); 0.671 | 0.79 (0.51-1.23); 0.301 |
| Dairy herd | 1.77 (0.97-3.21); 0.061 | 2.37 (1.25-4.47);  0.008 | 2.37 (1.23-4.56); 0.010 |
| Herd size | 0.99 (0.97-3.21); 0.886 | 0.96 (0.85-1.09); 0.572 | 0.98 (0.87-1.11); 0.738 |
| Farm area | 17.23 (5.63-52.66)  <0.001 | 22.21 (7.33-67.28);  <0.001 | 34.95 (10.31-118.41);  <0.001 |
| Confirmed historic incidence | 1.13 (0.79-1.62); 0.512 | 1.14 (0.79-1.66); 0.480 | 1.06 (0.73-1.53); 0.778 |
| Number of tested, unrestricted herds in the previous two years | 0.87 (0.62-1.21); 0.395 | 0.84 (0.54-1.29); 0.416 | 0.76 (0.45-1.26); 0.286 |
| Negative log likelihood | 107.85 | 101.41 | 100.87 |
| Degrees of freedom | 213 | 213 | 213 |

*These results were previously published by Vial and Donnelly (2012) in Supplemental Table 12.
