# Supplementary material for: Localised Badger Culling Increases Risk of Herd Breakdown on Nearby, Not Focal, Land
Source: PLoS One. 2016 Oct 17;11(10):e0164618. doi: 10.1371/journal.pone.0164618 (PMC5066978; doi:10.1371/journal.pone.0164618)
Supplement: S2 Table — Estimated odds ratios and their confidence intervals correspond to the change in risk of herd breakdown associated with a doubling of that variable. Negative log likelihood of the model = 91.11, d.f. = 207. (DOCX) [file pone.0164618.s004.docx]

| Variable | Odds ratio (95% confidence limit); p-value |
| --- | --- |
| Number of Badgers Culled <1km in the previous two years | 0.93 (0.80-1.08); 0.356 |
| **Number of Badgers Culled 1-3km in the previous two years** | **1.20 (1.01-1.43); 0.034** |
| **Number of Badgers Culled 3-5km in the previous two years** | **1.40 (1.16-1.69); <0.001** |
| Number of confirmed herd breakdowns  <1km in the previous two years | 1.69 (1.10-2.54); 0.017 |
| Number of confirmed herd breakdowns 1-3km in the previous two years | 0.80 (0.45-1.40); 0.432 |
| Number of confirmed herd breakdowns 3-5km in the previous two years | 0.79 (0.47-1.33); 0.371 |
| Dairy herd | 2.28 (1.13-4.61); 0.022 |
| Herd size | 0.97 (0.85-1.11); 0.673 |
| Farm area | 36.26 (9.30-141.42); <0.001 |
| Confirmed historic incidence | 1.09 (0.73-1.64); 0.662 |
| Number of tested, unrestricted herds <1km in the previous two years | 0.83 (0.55-1.25); 0.369 |
| Number of tested, unrestricted herds 1-3km in the previous two years | 1.23 (0.67-2.26); 0.497 |
| Number of tested, unrestricted herds 3-5km in the previous two years | 0.77 (0.39-1.52); 0.452 |
