# Supplementary material for: Localised Badger Culling Increases Risk of Herd Breakdown on Nearby, Not Focal, Land
Source: PLoS One. 2016 Oct 17;11(10):e0164618. doi: 10.1371/journal.pone.0164618 (PMC5066978; doi:10.1371/journal.pone.0164618)
Supplement: S3 Table — Results are presented of analyses based on data quantified, where appropriate, within a 1km radius, a 1-3km annulus, and a 3-5km annulus of the case-control. Estimated odds ratios and their confidence intervals correspond to the change in risk of herd breakdown associated with a doubling of that variable. (DOCX) [file pone.0164618.s005.docx]

|  | Odds ratio (95% confidence interval); p-value | | |
| --- | --- | --- | --- |
| Variable | <1km | 1-3km | 3-5km |
| **Number of badgers culled per km^2^ in the previous year** | **1.28 (1.13-1.44); <0.001** | **2.02 (1.58-2.59); <0.001** | **2.31 (1.68-3.20); <0.001** |
| Number of confirmed herd breakdowns in the previous year | 1.23 (0.96-1.60); 0.097 | 0.83 (0.56-1.24); 0.374 | 0.76 (0.50-1.17); 0.212 |
| Dairy herd | 1.97 (1.07-3.66); 0.030 | 2.37 (1.25-4.49); 0.008 | 2.26 (1.20-4.25); 0.012 |
| Herd size | 0.97 (0.86-1.10); 0.64 | 0.95 (0.84-1.09); 0.480 | 0.95 (0.84-1.07); 0.381 |
| Farm area | 23.57 (7.62-72.97); <0.001 | 22.96 (7.23-72.87); <0.001 | 30.89 (9.50-100.43); <0.001 |
| Confirmed historic incidence | 0.94 (0.51-1.73); 0.844 | 0.88 (0.45-1.76); 0.726 | 0.80 (0.39-1.62); 0.531 |
| Number of tested, unrestricted herds in the previous year | 0.83 (0.65-1.06); 0.132 | 0.83 (0.60-1.15); 0.271 | 0.91 (0.67-1.25); 0.571 |
| Negative log likelihood | 103.63 | 95.54 | 102.18 |
| Degrees of freedom | 213 | 213 | 213 |
