# Supplementary material for: Localised Badger Culling Increases Risk of Herd Breakdown on Nearby, Not Focal, Land
Source: PLoS One. 2016 Oct 17;11(10):e0164618. doi: 10.1371/journal.pone.0164618 (PMC5066978; doi:10.1371/journal.pone.0164618)
Supplement: S4 Table — Estimated odds ratios and their confidence intervals correspond to the change in risk of herd breakdown associated with a doubling of that variable. Negative log likelihood of the model = 90.68, d.f. = 207. (DOCX) [file pone.0164618.s006.docx]

| Variable | Odds ratio (95% confidence limit); p-value |
| --- | --- |
| Number of badgers culled per km^2^ <1km in the previous year | 0.98 (0.83-1.17); 0.856 |
| **Number of badgers culled per km^2^ from**  **1-3km in the previous year** | **1.66 (1.17-2.36); 0.005** |
| **Number of badgers culled per km^2^ from**  **3-5km in the previous year** | **1.63 (1.12-2.38); 0.012** |
| Number of confirmed herd breakdowns  <1km in the previous year | 1.26 (0.94-1.69); 0.119 |
| Number of confirmed herd breakdowns  1-3km in the previous year | 0.81 (0.52-1.27); 0.372 |
| Number of confirmed herd breakdowns  3-5km in the previous year | 0.81 (0.49-1.35); 0.426 |
| Dairy herd | 2.36 (1.19-4.69); 0.014 |
| Herd size | 0.94 (0.83-1.08); 0.411 |
| Farm area | 29.62 (8.53-102.88); <0.001 |
| Confirmed historic incidence | 0.81 (0.38-1.75); 0.603 |
| Number of tested, unrestricted herds <1km in the previous year | 0.88 (0.63-1.23); 0.472 |
| Number of tested, unrestricted herds 1-3km in the previous year | 0.83 (0.49-1.42); 0.507 |
| Number of tested, unrestricted herds 3-5km in the previous year | 1.07 (0.65-1.75); 0.782 |
